# Evaluation of Hematological Parameters as Markers for Subclinical Inflammation in Adults with Familial Mediterranean Fever

**DOI:** 10.34172/mejdd.2024.399

**Published:** 2024-10-30

**Authors:** Mohamed Gamal Abd Rabou, Ali Mahmoud Ramadan, Ahmed Mohamed Mohsen, Marwa Shawky

**Affiliations:** ^1^Department of Tropical Medicine, Faculty of Medicine, Alexandria University, Alexandria, Egypt; ^2^Department of Internal Medicine, Faculty of Medicine, Alexandria University, Alexandria, Egypt; ^3^Department of Epidemiology, High Institute of Public Health, Alexandria University, Alexandria, Egypt

**Keywords:** Familial Mediterranean fever, Red blood cell distribution width, Mean platelet volume, Neutrophil to leukocyte ratio, Platelet to lymphocyte ratio

## Abstract

**Background:**

Repeated polyserositis, another name for familial Mediterranean fever (FMF), is an autoimmune disorder with an autosomal recessive nature primarily characterized by short-lived repeated periods of peritonitis, pleuritis, and arthritis, generally accompanied by fever.

**Methods:**

Our participants were divided into two groups. Group I (patients): 100 individuals who were diagnosed as patients with FMF and were monitored. Group II (control): matched- healthy individuals (100 controls). They were compared and followed up as regards demographic, clinical, and laboratory data: routine investigations, neutrophil/lymphocyte ratio (NLR), platelet lymphocyte ratio (PLR), and mean platelet volume (MPV), red cell distribution width (RDW), C-reactive protein (CRP), and erythrocyte sedimentation rate (ESR).

**Results:**

Group I: MPV mean was 12.03±2.89, whereas group II MPV mean was 7.74±0.57. MPV was significantly statistically greater in group I than in group II. RDW mean in group I was 17.07±1.39 and in group II was 12.92±0.63. RDW was also significantly statistically greater in group I compared with group II. Group I’s NLR mean was 3.05±0.71, whereas group II’s NLR mean was 1.75±0.2. PLR mean in group I was 164.8±122.8 and in group II was 111.26±29.16.

**Conclusion:**

A statistically significant association was shown between the diagnosis of adult FMF and NLR, PLR, MPV, and RDW.

## Introduction

 Repeated polyserositis, another name for familial Mediterranean fever (FMF), is an autoimmune disorder with an autosomal recessive nature primarily characterized by short-lived repeated periods of peritonitis, pleuritis, and arthritis, generally accompanied by fever. As the name suggests, FMF is a family-centered condition that primarily affects people of Mediterranean origin.^1^

 Episodes of FMF are characterized by high fever, generally lasting from a few hours to a few days, as well as 90% serositis, fever, 33% arthritis, 31% pleuritis, 5% scrotum pain, and 1% pericardium. The rash that resembles erysipelas is typically linked to arthritis and affects the back of the proximal foot near the ankle joint as well as the distal end of the lower extremities, typically between the knee and the ankle joints. Patients are perfectly well in between FMF episodes.^2,3^

 Despite being more common in persons of Mediterranean descent, FMF is seen all over the world as a result of the 20th century’s massive demographic shifts. When it comes to adulthood, the men-to-women ratio is 1.5-2:1. By the time of diagnosis, 5%–10% of individuals with FMF are older than 20, 80%–95% are younger than 20, and 50% are younger than 10 years old. It is uncommon for individuals over 40 to experience symptoms. Of the 18 heterozygous children with onset before age 6, five were able to discontinue using colchicine before puberty and enter remission, according to a retrospective analysis.^4^

 Neutrophil extracellular traps (NETs) filaments of chromatin “decorated” with neutrophil granules and proteins of cytoplasm, including functional IL-1βare another hallmark of FMF attack. The self-limiting nature of FMF attacks may be explained by the negative feedback mechanism that NETs use to limit their development.^5,6^

 It is assumed that individuals with FMF experience inflammatory episodes that result in an acute phase of excessive creation of amyloid A serum that is reactive and contains proteins amyloid A, which subsequently deposits in both kidneys. However, amyloidosis only strikes those with special haplotypes of Mediterranean fever MEFV.^7^

 The gene MEFV, which is found on chromosome 16 at its short arm, is linked to missense and non-sense mutations that cause FMF, a recessive genetic disorder. The pyrin protein, often named marenostrin, is encoded by this gene. The MEFV gene contains more than 310 sequence variations, not all of which are linked to a specific disease phenotype.^6^

 Systemic inflammation can be detected by the measures of mean platelet volume (MPV), red cell distribution width (RDW), platelet count-to-absolute lymphocyte ratio (PLR), and absolute neutrophil-to-absolute lymphocyte ratio (NLR). Hepatic cirrhosis, ulcerative colitis, cancer, cardiovascular disorders, and systemic lupus erythematosus have all been linked to these parameters. Additionally, studies have shown that individuals with FMF have considerably greater NLR and MPV.^8-10^

 The aim of the current work was to ascertain if there is a relationship between RDW levels and FMF, explore the possibility of using MPV as inflammatory indicators in FMF patients, and explore the potential use of NLR, PLR, RDW, and MPV in the identification of inflammatory subclinical associated with FMF.

## Materials and Methods

 Two groups were involved in this investigation as a case-control study: Group I (patients): 100 adult patients with an FMF diagnosis were tracked down. Group II (controls): 100 age- and sex-matched healthy people. The patients’ genetic, clinical, and laboratory data were collected in a standardized form and retrospectively retrieved from the hospital files.

 The following details were also noted: sex, age, weight, height, presence of fever upon diagnosis, consanguineous marriage, family history of FMF, stomach discomfort, arthritis pain, investigation into the connection between such factors, and mutation in the genetics. FMF was diagnosed using the Tel-Hashomer criteria: the presence of a minimum of one of the four main criteria, a pair of the five minor criteria, five of the ten supporting criteria plus one minor criterion, or four of the five specific supportive criteria.

 When the following conditions were satisfied, an FMF attack was identified:

1. Applying in the first 72 hours after developing clinical symptoms (fever, abdominal discomfort, chest pain, arthralgia, pleuritis, serositis, pericarditis, arthritis, peritonitis, myalgia, and erythema mimicking erysipelas).
2. Excluding any further reasons for the fever.
3. A fever should continue for no less than 12 hours and be greater than 37 °C.
4. The following laboratory results are present: fibrinogen ≥ 350 mg/h, erythrocyte sedimentation rate (ESR) > 30 mm/h, C-reactive protein (CRP) ≥ 5 mg/dL, and white blood cell (WBC) count ≥ 10 000/mm^3^.

▪ Patients’ clinical and laboratory findings during the FMF attack have been recorded.
▪ The free attack period was considered to be at least two weeks from the completion of the last FMF attack.

###  Exclusion Criteria

 Those who have splenomegaly, diabetes mellitus, asthma, hematologic diseases, liver or renal insufficiency, uncontrolled blood pressure, a proteinuric state, and individuals who were prescribed non-steroidal anti-inflammatory medications or anticoagulant treatment.

###  Laboratory Analyses

- Hemoglobin, ESR, CRP, fibrinogen, serum electrolytes, blood sugar, urea, and tests of liver function were examined both during the attack and at least a month after it started.
- An automated blood count instrument was used to analyze the hemograms. The hemogram findings were used to record the following values: WBC, neutrophil count (K/μL), lymphocyte count (K/μL), platelet count (K/μL), NLR, PLR, MPV (fL), RDW, CRP, and ESR.
- The MEFV gene’s exon 2 and exon 10 mutation frequencies were identified for each patient using the DNA sequencing approach.
- Every blood sample was examined using an identical, often inspected analyzer (Abbott CELL-DYN 3700, USA).

## Results

###  Sociodemographic Characteristics of the Studied Group

 Groups I and II had mean ages of 22.31 ± 3.66 and 22.4 ± 3.49, respectively. Comparing both groups, there was no statistically significant variation. Group I’s average weight was 71.89 ± 4.51 kg, whereas group II’s mean weight was 69.46 ± 5.27 kg. The two groups differed statistically in a significant way. Furthermore, group I’s mean body mass index (BMI) was 24.76 ± 1.79, whereas group II’s was 23.72 ± 2.14. Regarding BMI, a significant statistical distinction was seen between both groups (Table 1).

**Table 1:** Sociodemographic characteristics of the studied group

	**Patients with FMF (n=100)**	**Well-being controls (n=100)**	***P***
Age (y)			0.859
Minimum–Maximum	18.0 – 34.0	18.0 – 33.0	
Mean ± standard deviation	22.31 ± 3.66	22.4 ± 3.49	
Sex			0.671
Male	47 (47.0)	50 (50.0)	
Female	53 (53.0)	50 (50.0)	
Height (cm)			0.121
Minimum–Maximum	165.0 – 179.0	165.0 – 180.0	
Mean ± standard deviation	170.49 ± 3.1	171.28 ± 4.03	
Weight (kg)			0.001^*^
Minimum–Maximum	60.0 – 83.0	58.0 – 81.0	
Mean ± standard deviation	71.89 ± 4.51	69.46 ± 5.27	
BMI			< 0.001*
Minimum–Maximum	20.24 – 29.38	19.05 – 28.36	
Mean ± standard deviation	24.76 ± 1.79	23.72 ± 2.14	

BMI, body mass index. *Significat *P* value

###  Clinical History of Patients with FMF

In FMF group; the age of diagnosis ranged from 5.0–17.0 years, with mean of 10.64 ± 2.95 years. 100% of FMF group suffered from fever, while chest pain, abdominal pain, muscle pain, joint pain, skin rash were present in 82%, 65%, 46%, 55%, 16%, respectively. 57% of the cases showed positive family history while 15 % showed Presence of consanguinity (Table 2).

**Table 2:** Clinical history of patients with FMF

	**Patients with FMF (n=100)**
Age in years at diagnosis	
Minimum–Maximum	5.0 – 17.0
Mean ± standard deviation	10.64 ± 2.95
Clinical symptoms	
Fever	100 (100)
Chest pain	82 (82.0)
Abdominal pain	65 (65.0)
Muscle pain	46 (46.0)
Joint pain	55 (55.0)
Skin rash	16 (16.0)
Positive family history	57 (57.0)
Presence of consanguinity	15 (15.0)

### Hematological Findings of the Studied Groups

 Red blood cells (RBCs), platelets, WBCs, and neutrophils were much greater in group I compared with group II. The MPV average for group I was 12.03 ± 2.89, whereas group II’s MPV mean was 7.74 ± 0.57. In comparison with group II, MPV was statistically and substantially greater in group I. The RDW mean for groups I and II was 17.07 ± 1.39 and 12.92 ± 0.63, respectively. RDW was also significantly greater in group I than in group II (Table 3).

**Table 3:** Hematological findings of the studied groups

	**Patients with FMF (n=100)**	**Healthy controls (n=100)**	***P***
RBCs (cell/cmm)			< 0.001*
Minimum–Maximum	3.5 – 5.5	4.0 – 5.5	
Mean ± standard deviation	4.72 ± 0.32	4.54 ± 0.27	
Hemoglobin (g/dL)			0.861
Minimum–Maximum	13.0 – 16.0	12.0 – 16.0	
Mean ± standard deviation	14.13 ± 0.75	14.11 ± 0.78	
Platelets (cell/μL)			< 0.001*
Minimum–Maximum	30.0 – 500.0	170.0 – 374.0	
Mean ± standard deviation	370.5 ± 82.91	259.82 ± 53.31	
WBCs (cell/μL)			< 0.001*
Minimum–Maximum	7.0 – 15.0	5.5 – 10.5	
Mean ± standard deviation	11.03 ± 1.66	7.41 ± 1.21	
Neutrophils (cell/cmm)			< 0.001*
Minimum–Maximum	4000.0 – 11000.0	3000.0 – 6000.0	
Mean ± standard deviation	7436.95 ± 1379.92	4187.8 ± 865.86	
Lymphocytes (cell/cmm)			0.173
Minimum–Maximum	1530.0 – 4,125.0	1500.0 – 3500.0	
Mean ± standard deviation	2531.25 ± 594.37	2421.4 ± 541.1	
MPV(fL)			< 0.001*
Minimum–Maximum	7.0 – 16.0	7.0 – 9.0	
Mean ± standard deviation	12.03 ± 2.89	7.74 ± 0.57	
RDW (%)			< 0.001*
Minimum–Maximum	12.0 – 19.0	12.0 – 14.0	
Mean ± standard deviation	17.07 ± 1.39	12.92 ± 0.63	

*Significat *P* value

###  Laboratory Findings of the Studied Groups

 The fibrinogen mean for group I was 298.35 ± 54.02, whereas the mean for group II was 230.88 ± 20.57. Group I’s NLR mean was 3.05 ± 0.71, whereas group II’s NLR mean was 1.75 ± 0.2. PLR mean in group I was 164.8 ± 122.8, and in group II was 111.26 ± 29.16. Fibrinogen, NLR, and PLR were considerably and statistically more in group I compared with group II (Table 4).

**Table 4:** Laboratory findings of the studied sample

	**Patients with FMF (n=100)**	**Healthy controls (n=100)**	***P***
Fibrinogen (mg/dL)			< 0.001*
Minimum–Maximum	200.0 – 420.0	200.0 – 288.0	
Mean ± standard deviation	298.35 ± 54.02	230.88 ± 20.57	
ESR (mm/h)			< 0.001*
Minimum–Maximum	5.0 – 22.0	2.0 – 13.0	
Mean ± standard deviation	10.22 ± 2.7	8.7 ± 2.64	
CRP (mg/L)			< 0.001*
Minimum–Maximum	3.0 – 16.0	1.0 – 6.0	
Mean ± standard deviation	8.35 ± 3.01	3.74 ± 1.43	
Serum level of urea (mg/dL)			0.028*
Minimum–Maximum	14.0 – 40.0	16.0 – 41.0	
Mean ± standard deviation	27.57 ± 6.22	29.66 ± 7.12	
Serum level of creatinine (µmol/L)			0.097
Minimum–Maximum	0.4 – 1.3	0.4 – 1.3	
Mean ± standard deviation	0.87 ± 0.22	0.81 ± 0.22	
ALT (U/L)			0.002*
Minimum–Maximum	14.0 – 33.0	10.0 – 37.0	
Mean ± standard deviation	24.54 ± 5.58	21.74 ± 7.03	
AST (U/L)			< 0.001*
Minimum–Maximum	13.0 – 40.0	9.0 – 35.0	
Mean ± standard deviation	25.26 ± 6.26	20.58 ± 6.6	
FBS (mg/dL)			< 0.001*
Minimum–Maximum	76.0 – 109.0	77.0 – 100.0	
Mean ± standard deviation	94.16 ± 7.07	90.16 ± 6.54	
Na (mEq/L)			0.238
Minimum–Maximum	135.0 – 144.0	135.0 – 144.0	
Mean ± standard deviation	139.6 ± 2.42	139.2 ± 2.36	
K (mEq/L)			0.015*
Minimum–Maximum	3.5 – 5.2	3.5 – 5.0	
Mean ± standard deviation	4.23 ± 0.5	4.07 ± 0.42	
NLR			< 0.001*
Minimum–Maximum	1.6 – 4.68	1.31 – 2.0	
Mean ± standard deviation	3.05 ± 0.71	1.75 ± 0.2	
PLR			< 0.001*
Minimum–Maximum	48.96 – 1333.3	56.0 – 166.0	
Mean ± standard deviation	164.8 ± 122.8	111.26 ± 29.16	

*Significat *P* value

###  Level of Serum Amyloid A in Both Groups

 The mean level of serum amyloid A was 5.28 ± 1.44 among group I and 4.52 ± 1.01 among group II, indicating a substantial difference in serum amyloid A levels between the two groups (Table 5).

**Table 5:** Level of serum amyloid A in the examined sample

	**Patients with FMF (n=100)**	**Healthy controls (n=100)**	***P***
Serum amyloid A (mg/L)			< 0.001*
Minimum–Maximum	3.0 – 12.0	3.0 – 6.0	
Mean ± standard deviation	5.28 ± 1.44	4.52 ± 1.01	

*Significat *P* value

###  NLR, PLR, MPV, and EDW Diagnostic Performance in the Evaluation of FMF

**Figure 1:**
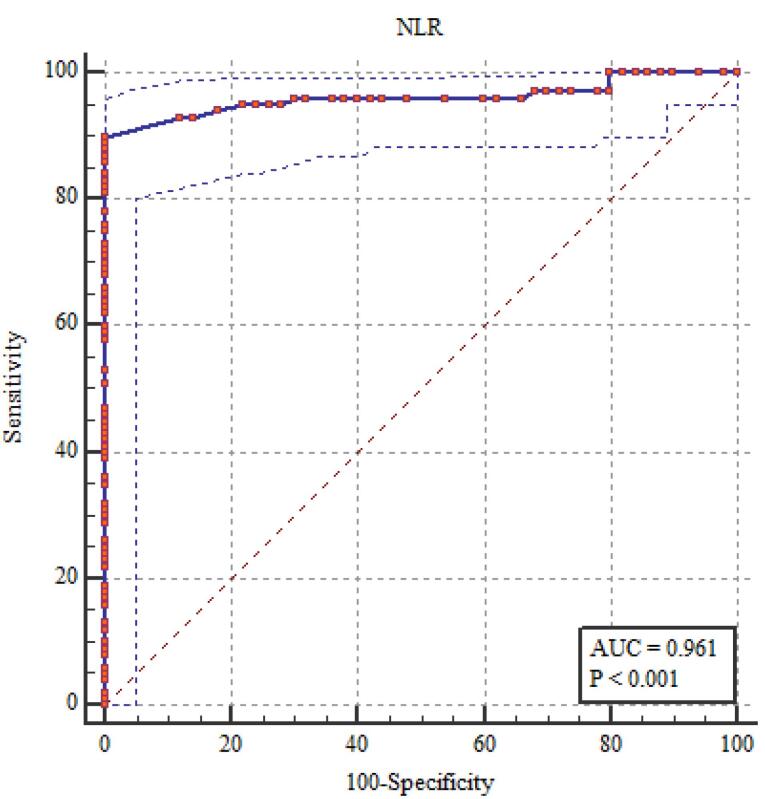
ROC curve of NLR

**Figure 2:**
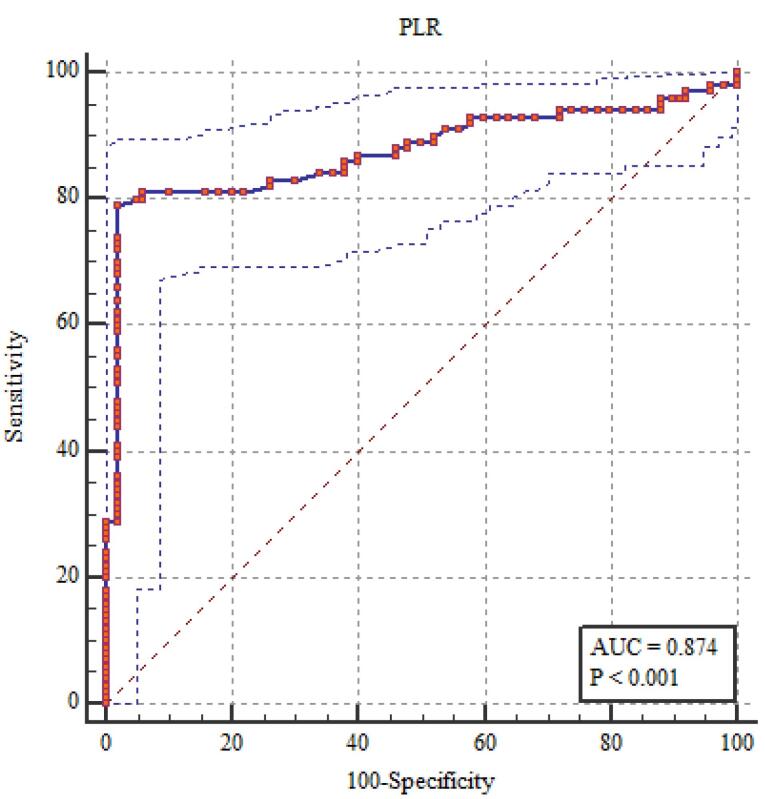
ROC curve of PLR

**Figure 3:**
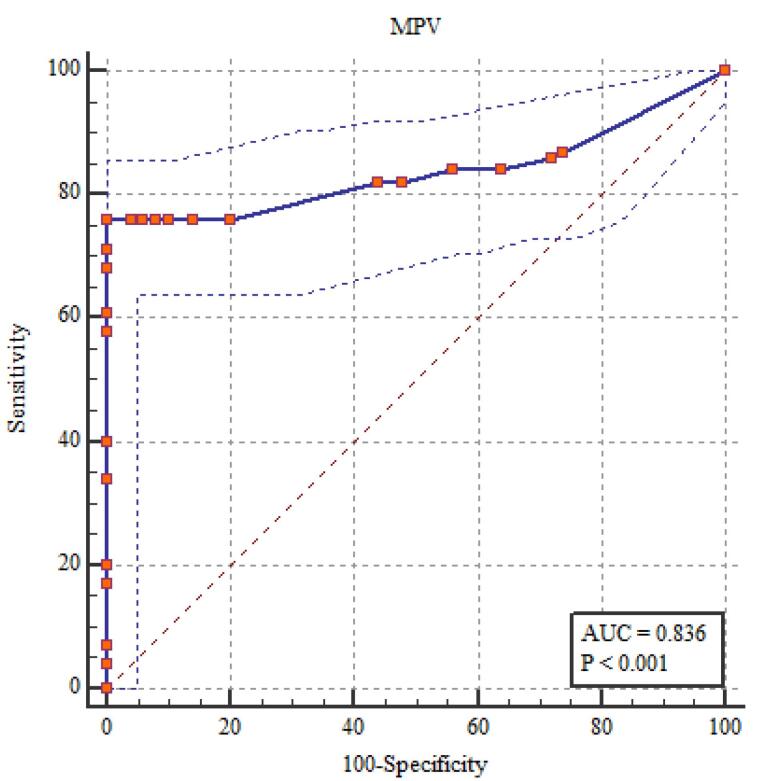
ROC curve of MPV

**Figure 4:**
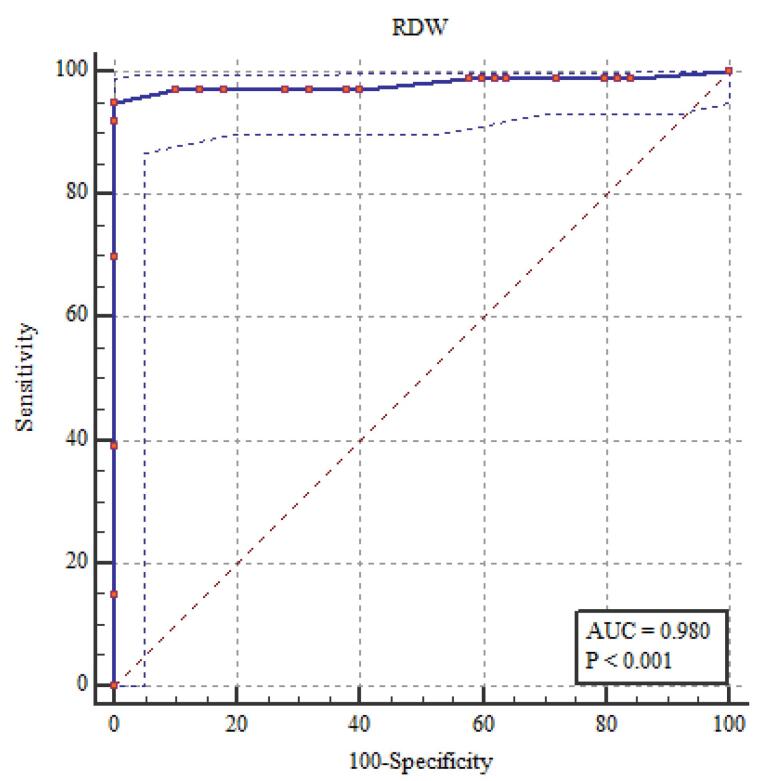
ROC curve of RDW

**Figure 5:**
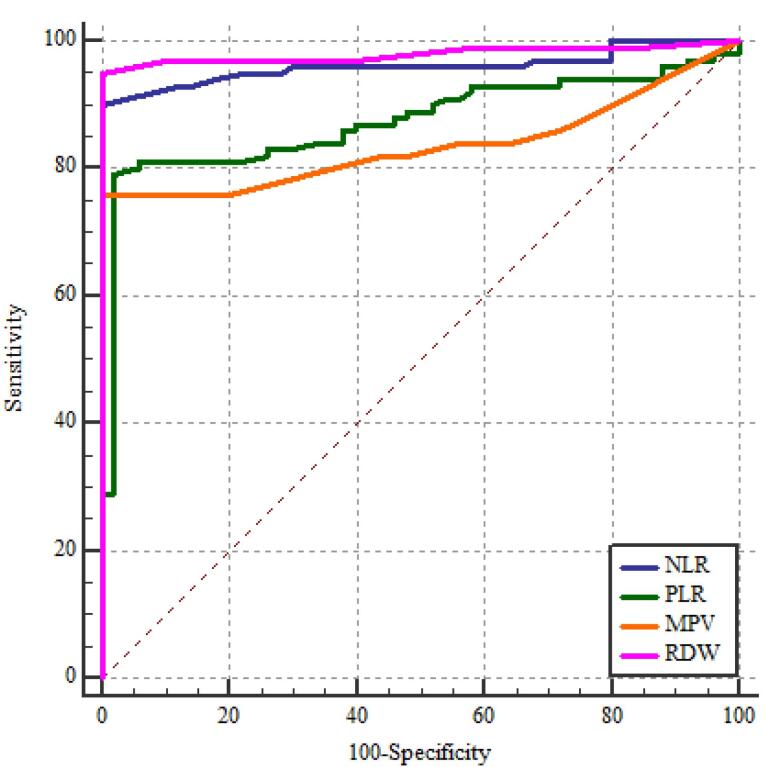
ROC curve of NLR, PLR, MPV, and RDW

 It showed statistically significant correlations between the diagnosis of FMF and RDW, NLR, PLR, and MPV. The most sensitive relation was between RDW and NLR (Table 6; Figures 1-5).

**Table 6:** NLR, PLR, MPV, and EDW diagnostic performance in the evaluation of FMF

	**The area under the curve (AUC) (95% CI)**	**Cut off point**	**Sensitivity percent (95% CI)**	**Specificity percent (95% CI)**	***P***
NLR	0.961 (0.924 - 0.983)	> 2.0	90.0 (82.4 - 95.1)	100 (96.4 - 100)	< 0.001*
PLR	0.874 (0.820 - 0.917)	> 150.0	79.0 (69.7 - 86.5)	98.0 (93.0 - 99.8)	< 0.001*
MPV(fL)	0.836 (0.778 – 0.885)	> 9.0	76.0 (66.4 – 84.0)	100 (96.4 – 100)	< 0.001*
RDW (%)	0.980 (0.950 - 0.995)	> 14.0	95.0 (88.7 - 98.4)	100 (96.4 - 100)	< 0.001*

## Discussion

 The most harmful FMF consequence is amyloidosis. Despite treatment, subclinical inflammation is the primary cause of amyloidosis in patients with FMF. In FMF, several inflammatory markers have been investigated. FMF acute phase response is assessed using WBC levels, serum amyloid A protein, fibrinogen, ESR, and CRP levels as indicators.^11^

 In line with previous research, we discovered that patients with FMF had considerably greater levels of fibrinogen, ESR, serum amyloid A, and CRP in comparison with healthy individuals. These findings suggest that subclinical inflammation is occurring in patients with FMF. Up to 30% of patients with FMF have been shown to be still experiencing subclinical inflammation. A sustained increase in these markers is significant because it represents the subclinical inflammation that is primarily responsible for the onset of amyloidosis and its associated consequences, such as splenomegaly, anemia, and osteopenia.^12,13^

 One essential protein for the blood clotting process is fibrinogen. A routinely ordered non-specific test for the identification of inflammatory disorders is the ESR. One element of the acute phase response to both chronic as well as acute inflammation is a rise in CRP.^14^

 The current study found that elevated levels of fibrinogen, ESR, CRP, and amyloid A in patients with FMF indicated alterations in subclinical inflammation, as shown by MPV, RDW, NLR, and PLR. Among these indicators, NLR and RDW correlated most strongly with subclinical inflammation. These findings imply that NLR and RDW might help identify subclinical inflammation in FMF. By dividing the number of neutrophils by the number of lymphocytes, NLR—which was discovered to be connected to systemic inflammation is calculated.^12,15^

 In this study, group I’s NLR mean was 3.05 ± 0.71, while group II’s was 1.75 ± 0.2. Group I’s NLR was significantly greater than group II’s. Similar to what we found NLR in patients with FMF and healthy controls was studied by Uslu et al. They discovered that patients with FMF had considerably greater NLR. Additionally, they discovered that, in comparison with individuals without amyloidosis, patients with the disease had a considerably greater NLR.^12^

 According to Ahsen et al, patients with FMF may benefit from using NLR as an acute phase response.^16^ Uluca et al discovered that patients in the attack-free phase had greater NLR levels, and they concluded that NLR may be a sign of the attack period in patients with FMF.^17^ NLR levels of juvenile FMF patients without symptoms and healthy controls were examined by Özer et al. They discovered that NLR and CRP exhibited a strong association, leading them to the conclusion that in patients with FMF, NLR may be a useful indicator of subclinical inflammation.^18^

 Additionally, Uslu et al showed that individuals with FMF exhibited a higher NLR over the healthy controls during the attack-free interval. The NLR is a measure of subclinical inflammation and might be obtained by performing a full blood count.^12^

 As stated by Celikbilek et al, the NLR of adult patients with FMF during acute attacks and attack-free times was significantly higher during acute attack episodes compared with attack-free episodes and the control group.^19^

 The results we obtain offer only a small amount of evidence in favor of the theory that NLR might be a measure of inflammation in patients with FMF. Given its affordability, accessibility, and ease of calculation, NLR is an attractive option for predicting systemic inflammation in adult patients with FMF.

 The term RDW describes how the erythrocytes in the blood can fluctuate in size. In our investigation, we discovered that RDW levels were connected to subclinical inflammation. It also reflects the degree of inflammation.

 In the current research, we discovered that patients with FMF had considerably greater RDW levels than controls. In autoimmune diseases, a higher RDW has been associated with poorer clinical outcomes, according to research findings.^20^

 It has also been shown that there is a high association between RDW and commonly utilized inflammatory indicators like CRP and ESR.^21^

 According to Förhécz et al, there was a substantial positive association between CRP and RDW. It was discovered that individuals with heart failure have a negative acute-phase reactant (pre-albumin) and RDW relation that is very significant.^22^

 RDW was correlated with ESR and CRP in random outpatient adults, according to recent research by Lippi et al,^21^ Erdem et al, however, it was shown that individuals with inflammatory diseases, including reactive systemic AA amyloidosis, have lower MPVs and greater RDWs.^23^

 Yildirim Cetin et al have looked at the connection between RDW levels in patients with FMF to identify inflammatory disorders and make therapy options. Similar to our research, they concluded that patients with FMF may benefit from knowing about persistent subclinical inflammation if their RDW levels are high.^24^

 During a regular blood count, a measure called MPV is found that represents platelet function and activation.^25^ In the current research, the FMF patients’ MPV was statistically and considerably greater than that of the normal control group, which is consistent with many of the research findings. This suggests that MPV may be useful in demonstrating the likelihood of subclinical inflammation in FMF-affected adults.

 In the first investigation on this subject, Makay et al discovered that patients with FMF had lower MPV levels during an attack than healthy controls.^26^ These results were in disagreement with what we found in our search.

 Arica et al discovered that MPV was considerably greater in patients who had experienced an acute attack or a period of free attacks than in well-being controls. That is in line with our findings.^27^

 Furthermore, MPV levels were greater in patients with FMF throughout the free attacks period compared with well-being controls, according to Coban and Adanir’s findings.^28^

 Özer et al discovered that during the inter-attack interval, MPV was considerably greater in patients with FMF than in controls. They concluded that in patients with FMF, MPV might be a potential marker of subclinical inflammation.^18^

 The limitation of our study was the short follow-up period, so a larger study group is recommended. Thus, our results may provide a baseline for understanding the importance of the evaluation of hematological parameters as markers for subclinical inflammation in adults with FMF

## Conclusion

 A statistically significant association has been shown between the diagnosis of adult FMF and NLR, PLR, MPV, and RDW. RDW and NLR had the most sensitive association.

## Recommendations

1. According to our research findings:
  - In patients with FMF, MPV may be utilized as an inflammatory marker.
  - NLR, PLR, RDW, and MPV may be useful in identifying subclinical FMF inflammation.
  - RDW and NLR had the most sensitive association with subclinical inflammation in patients with adult FMF.
2. More research is required to evaluate the reliability of these factors.
